# Identification of MSA-2: An oral antitumor non-nucleotide STING agonist

**DOI:** 10.1038/s41392-020-00459-2

**Published:** 2021-01-12

**Authors:** Jianhua Liu, Xu Huang, Jianxun Ding

**Affiliations:** 1 Department of Urology, The First Hospital of Jilin University, 71 Xinmin Street, 130021 Changchun, People’s Republic of China; 2 Key Laboratory of Polymer Ecomaterials, Changchun Institute of Applied Chemistry, Chinese Academy of Sciences, 5625 Renmin Street, 130022 Changchun, People’s Republic of China; 3 Department of Hepatobiliary and Pancreatic Surgery, The First Hospital of Jilin University, 71 Xinmin Street, 130021 Changchun, People’s Republic of China

**Keywords:** Drug screening, Target validation

**Fig. 1:**
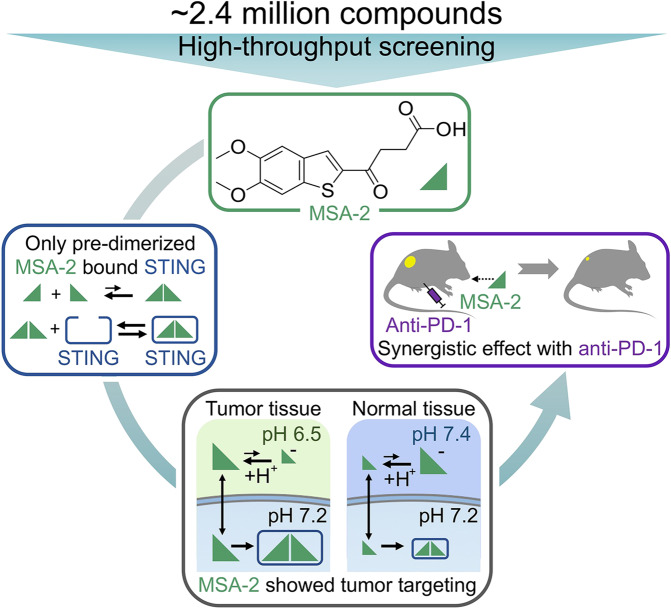
Through high-throughput screening, MSA-2 was identified as a promising STING agonist from ~2.4 million compounds. Only pre-dimerized MSA-2 could bind to STING. In the acidic tumor microenvironment, uncharged MSA-2 showed a higher permeable ability and thus preferentially activated STING compared to normal tissues. Oral administration of MSA-2 exhibited excellent safety tolerance and synergetic antitumor effects with anti-PD-1 therapy in vivo

In a recent study in *Science*, Pan et al.^1^ identified an orally available non-nucleotide human stimulator of interferon genes (STING) agonist, MSA-2 (benzothiophene oxobutanoic acid), with excellent safety tolerance in vivo. The noncovalently tethered dimers of MSA-2 could bind STING with nanomolar affinity, and the acidic tumor microenvironment would substantially increase the cell entry and retention of MSA-2 and its dimerized interaction with STING, thus leading to a superior antitumor potency of combined MSA-2 and anti-PD-1 treatments. This work has encouraged further design and discovery of clinical human STING agonists for systematic cancer therapy (Fig. 1).

The success of immune checkpoint blockade therapy has significantly extended the survival of cancer patients, inspiring more efforts to explore other immune pathways that can be modulated pharmacologically to enhance the efficacies of immune checkpoint inhibitors (ICIs).^2^ As a sensor of 2′,3′-cyclic guanosine monophosphate-adenosine monophosphate (cGAMP) at the endoplasmic reticulum, STING undergoes conformational change and activation via binding to cGAMP.^3^ The activation of STING then induces increased expression of type I interferon through triggering downstream signals, leading to stimulation of robust antitumor immunity correlated with T cell infiltration. The first generation cyclic dinucleotide (CDN)-based STING agonist could induce inflammatory cytokines in both normal and tumor tissues, owing to its low selectivity. The combination of CDN-based STING agonists and ICIs demonstrated its therapeutic potentials in preclinical models, while the administration of STING agonists was only limited to intratumoral injection because of the omnipresent STING expression.^4^ Thus, the development of advanced STING agonists for systemic administration will significantly enhance their antitumor effectiveness clinically.

Recently, Ramanjulu et al.^5^ discovered a small molecule STING agonist that could be used for intravenous administration. The developed agonist not only resolved the challenge of drug administration but also extended the therapeutic potentials toward solid tumors. In this follow-up study, Pan et al.^1^ developed a unique STING agonist, MSA-2, that displayed tumor targeting and could be administrated orally.

To discover the optimal STING agonist, Pan et al. have developed a high-throughput screening strategy to identify cell-permeable STING agonists. After screening a library of around 2.4 million compounds, several molecules were identified, including MSA-2. MSA-2 was revealed to activate STING selectively and exhibited higher permeability than CDNs like MSA-1, a cGAMP analog. Furthermore, MSA-2 displayed no significant effect in binding assays against various receptors. MSA-2 has further shown a dose-dependent antitumor effect by intratumoral, subcutaneous, or oral administration in vivo. The antitumor effect and tolerability through oral medication were equal to or better than those of MSA-1.

The X-ray crystal structure of the STING-MSA-2 complex showed a closed conformation of STING. A four-stranded β-sheet atop two MSA-2 molecules and two α_2_ helices on the STING formed a smaller angle than in the open conformation, similar to the cGAMP complex. The two MSA-2 molecules were contacted *via* the π-π stacking effect and stacked against Tyr^167^ from each STING subunit. The bound MSA-2 also interacted with surrounding side chains to form noncovalent crosslinking between the protein homodimer and stabilized the closed conformation. In addition, Pan et al. identified the shift perturbations of benzothiophene ring protons on MSA-2 in aqueous solution, consistent with a reversible dimerization process, indicating that most bioactive MSA-2 in the aqueous buffer are dimers binding to STING. Then, compound 2, an MSA-2 analog, in which the nitrogen of the thiophene ring replaced sulfur, was used to assess the binding mechanism. When STING was incubated with compound 2 at a concentration insufficient to cause detectable binding, adding MSA-2 induced binding of both MSA-2 and compound 2 to STING. The finding suggested that these heterodimers could bind to STING competently with MSA-2. Therefore, the noncovalent dimer, MSA-2 homodimers, is the bioactive agent to bind and activate STING.

Based on the bioactivity of MSA-2 dimer, Pan et al. predicted that a stable compound dimer would be a potent agonist. To design the most optimal ligand, a computational method was developed to score thousands of tethered benzothiophene cores, and the replacement of both 5-methoxy groups with a propane linker was highly promising. Synthesized covalent dimer 3 could efficiently bind STING in a noncovalent pose, indicating that the critical interactions of the carboxylic acid and ketone with STING replicated that of MSA-2. After verifying the three-atom linking strategy, Pan et al. further investigated a series of additional modifications, including four and five-atom linkers, while none of them exhibited significant enhancement on the binding efficiency to STING. The X-ray crystallographic data confirmed that the oxobutanoic acid structure dominated the binding pose for the molecules. Thus, the specific benzothiophene confirmation is the basis for drug design to preserve the oxobutanoic acid interaction with STING.

Owing to the increasing amount of uncharged MSA-2 as decreasing pH, Pan et al. hypothesized that the acidic tumor microenvironment would promote MSA-2 entry and enhance potency. Through calculation, when the ratio of uncharged MSA-2 to charged MSA-2 ≥ 50, the alterative pH from 6 to 7.5 significantly affected the intracellular concentrations of monomeric and dimeric MSA-2. In addition, there was a higher concentration of MSA-2 in the tumor than that in other tissues. Furthermore, Pan et al. investigated the enhancement of MSA-2 on anti-PD-1 treatment on immunologically “cold” tumors, including colorectal MC38, colorectal CT26, melanoma B16F10, and lung LL-2 tumors. For each model, the combination of anti-PD-1 intraperitoneally and MSA-2 orally showed synergistic antitumor effect compared to each monotherapy, and both innate and adaptive immune responses driven by STING agonist contributed to tumor regression.

In summary, the study by Pan et al. identified an orally available STING agonist, MSA-2, through high-throughput screening. As a single medication, MSA-2 suppressed tumor with both innate and adaptive immune responses and was well tolerated. Toward immunologically “cold” tumors, the combination of MSA-2 and anti-PD-1 therapy outperformed monotherapy. Owing to the unique mechanism, MSA-2 exhibited a higher potency in the acidic tumor microenvironment, where the small molecule underwent noncovalent dimerization to form a bioactive ligand. It is believed that the powerful MSA-2 will encourage researchers to discover other human STING agonists.
